# Supplementary figures and images for: Human subcortical pathways automatically detect collision trajectory without attention and awareness
Source: PLoS Biol. 2024 Jan 18;22(1):e3002375. doi: 10.1371/journal.pbio.3002375 (PMC10795999; doi:10.1371/journal.pbio.3002375)

## a: Experiment 1

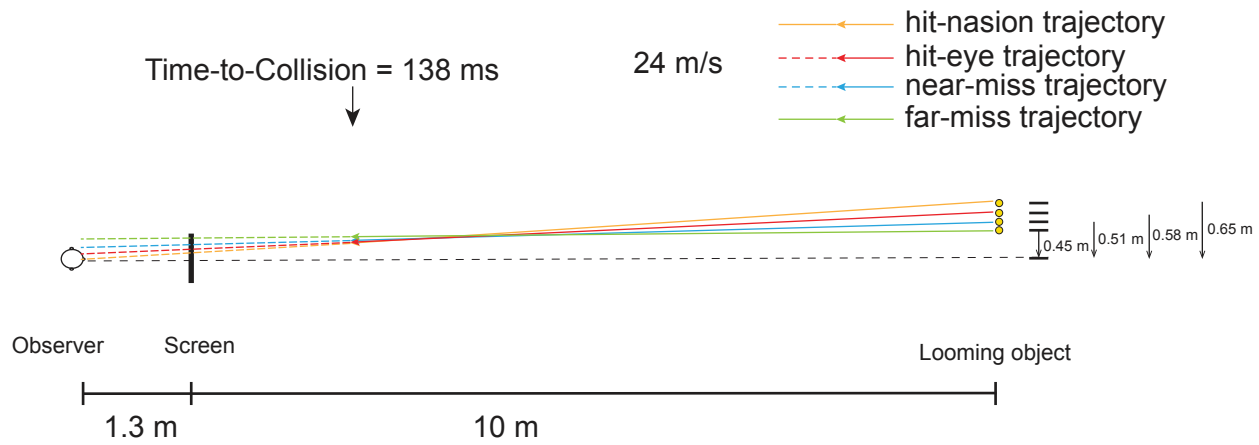

## b: Experiment 2

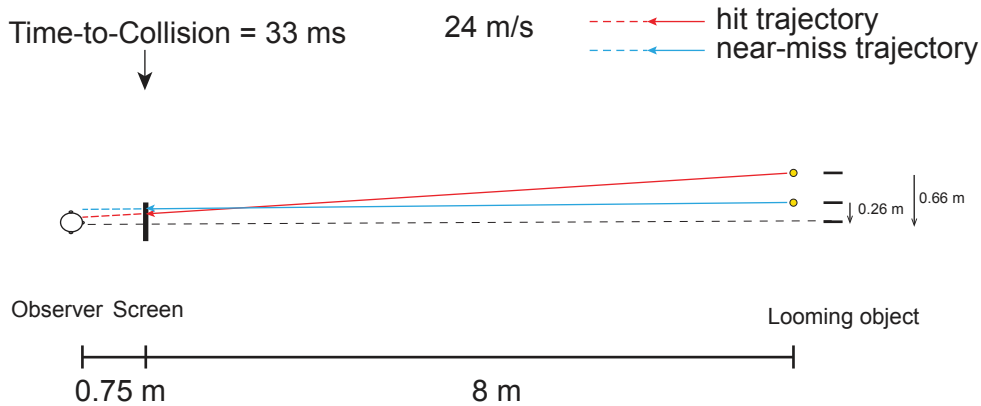

## c: Experiment 3

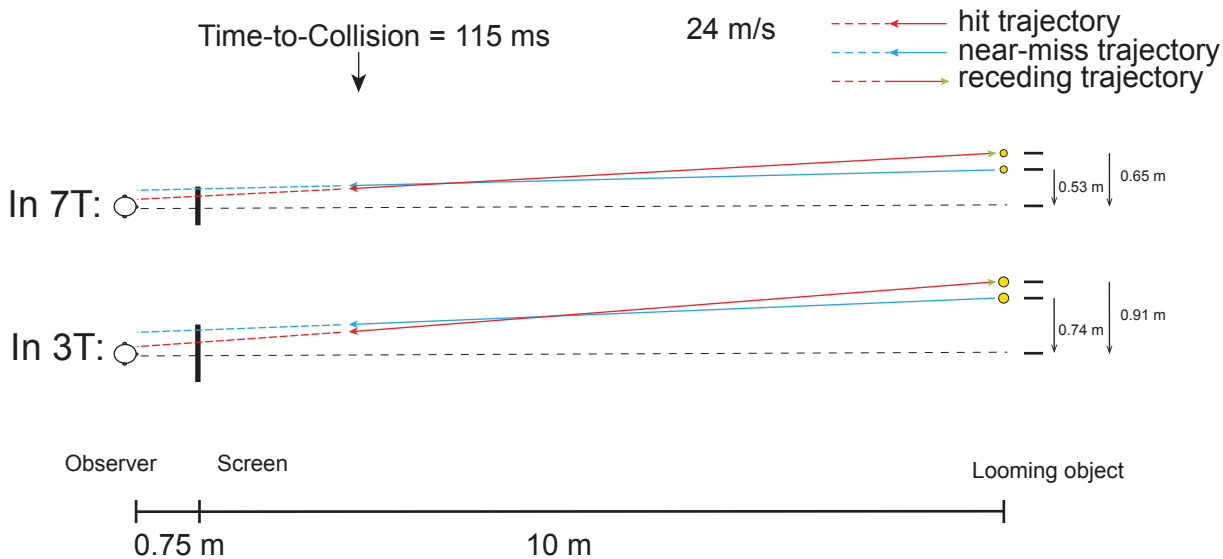

Supplement: S1 Fig — (a) Experiment 1. A baseball-sized sphere launched 11.3 meters away from the observer, moving at a speed of 24 m/s. It disappeared at 3.3 meters from the observers at 138 ms of time-to-collision, as indicated by the location of the arrows. Yellow, red, blue, and green arrows indicate the hit-nasion, hit-eye, near-miss, and far-miss trajectories. Stimuli were presented on a 3D monitor (width = 0.51 m) 1.3 meters in front of the observers. The on-screen display was a sphere expanding from 0.3 to 1 degree of visual angle in 330 ms. While the vertical offset of the stimulus from the center of screen was 1.90°, the horizonal offset for the starting (hit nasion: 3.14°, hit eye: 2.79°, near miss: 2.43°, far miss: 2.13°) and ending (hit nasion: 2.77°, hit eye: 2.79°, near miss: 2.80°, far miss: 2.86°) position of the stimulus varied between different trajectory conditions. (b) Experiment 2. Stimuli were presented on a translucent screen with a 2D projector. The sphere moved from 8.75 m away and disappeared on the screen (width = 0.35 m) 0.75 m in front of the observer at 33 ms of time-to-collision. Red and blue arrows indicate the hit and near-miss trajectories. The stimulus on the screen expanded from 0.4 to 4.5 degrees of visual angle in 330 ms. While the vertical offset of the stimulus from the center of screen was 2.49°, the horizonal offset for the starting (hit: 4.31°, near miss: 1.70°) and ending (hit: 4.31°, near miss: 5.87°) position of the stimulus varied between different trajectory conditions. (c) Experiment 3. In hit and near-miss conditions, the sphere moved from 10.75 m to 2.75 m in front of the observer. The time-to-collision at disappearance was 115 ms. The receding trajectory was the reverse of the hit trajectory. Red, blue, and green arrows indicate the hit, near-miss, and receding trajectories. The eccentricity and the size of the sphere (7T: 6 cm, screen width = 0.35 m; 3T: 8.4 cm, screen width = 0.51 m) was slightly different in the 7T and 3T scanning. [file pbio.3002375.s001.pdf]

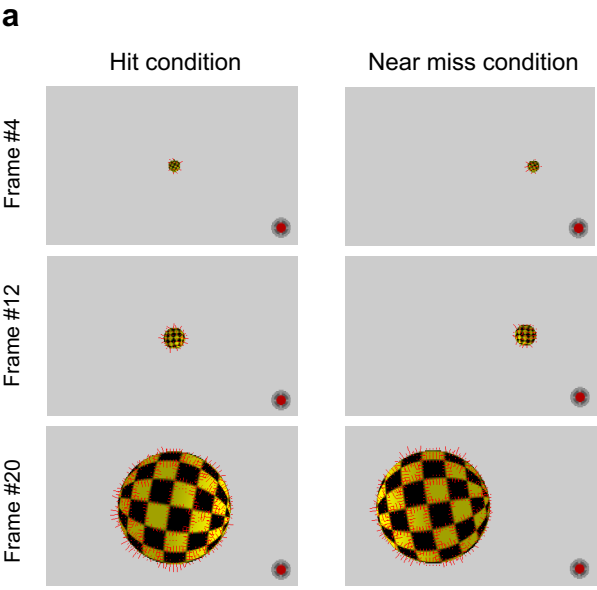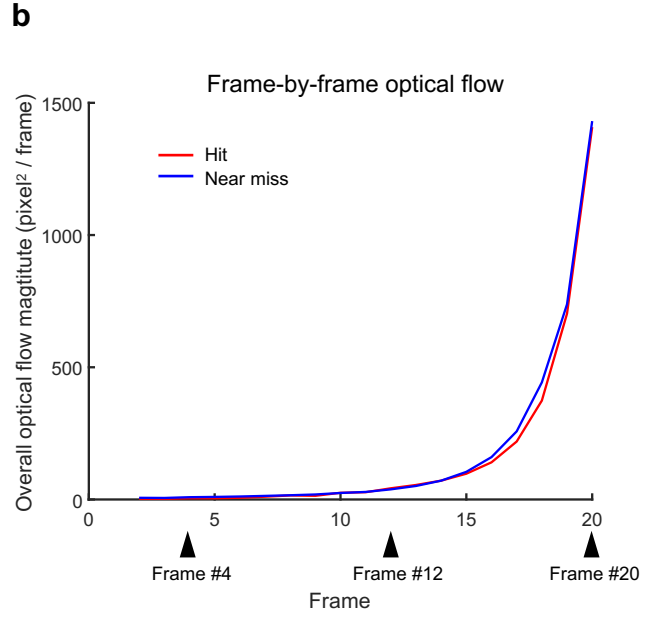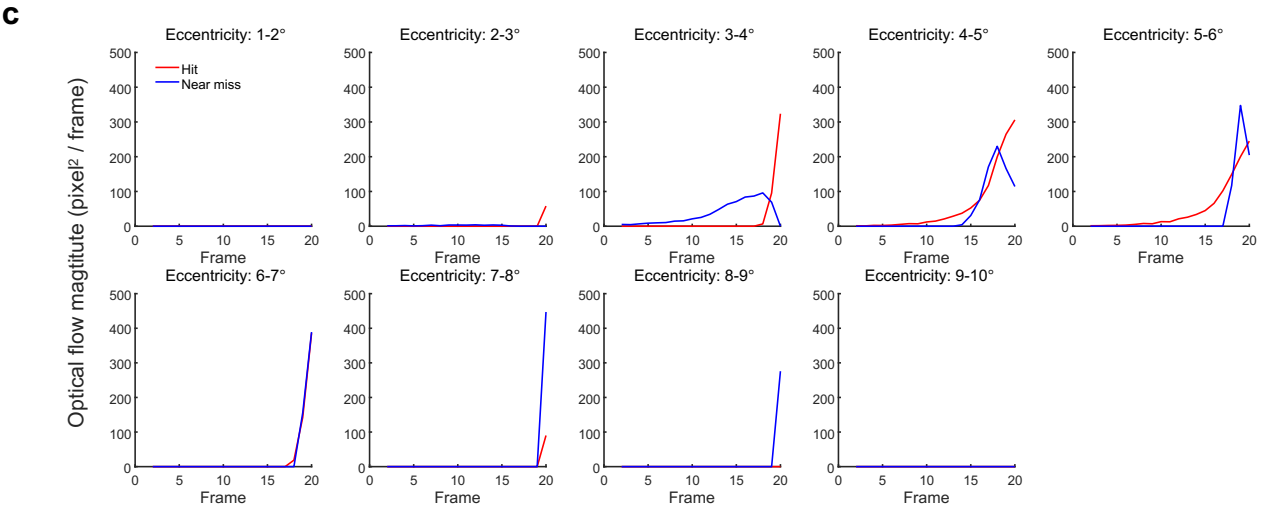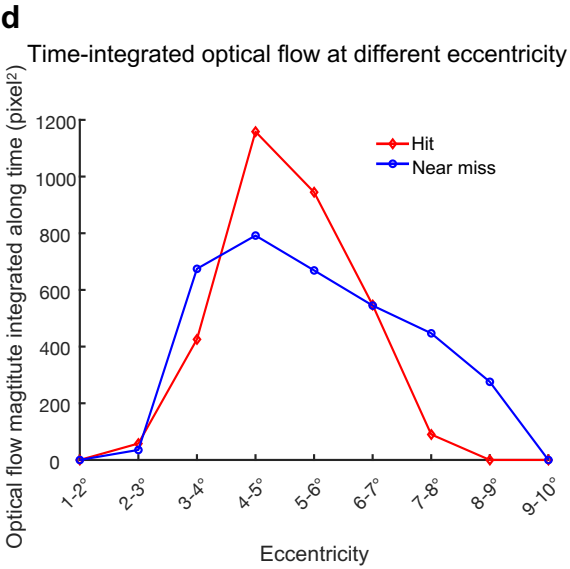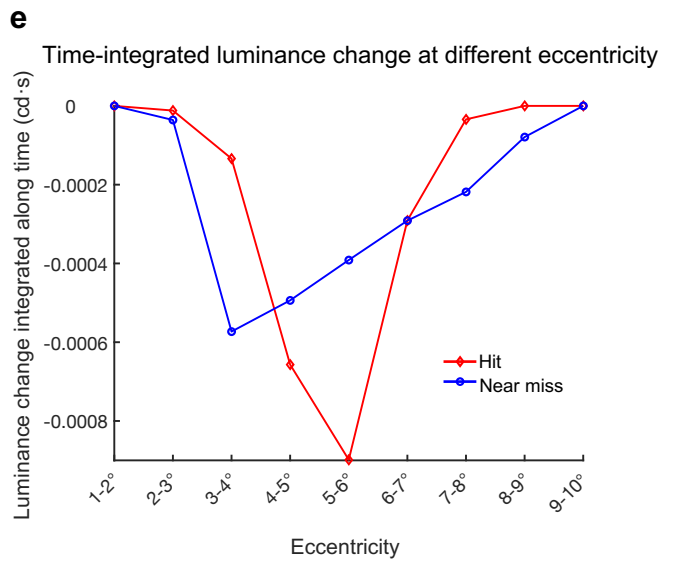

Supplement: S2 Fig — We used the Horn–Schunck method [74] to compute the frame-by-frame optical flow of our stimuli. The Horn–Schunck method is utilized to determine the optical flow by analyzing the displacement of pixels between frames, assuming that the brightness of a pixel remains constant during its motion. In our analysis, we captured the image in each frame during the visual stimulation. Subsequently, we employed the MATLAB Optical Flow block (the mathematical algorithm [74] is given in S1 Text) from the Computer Vision Toolbox to estimate the optical flow vector for each pixel between 2 consecutive frames. The direction of pixel motion was determined by the horizontal and vertical components of the vector, while the speed or magnitude was calculated as the square of the vector’s modulus. Finally, we computed the overall flow magnitude by summing the magnitudes of all the pixels. The scripts for this analysis have been uploaded to https://doi.org/10.5281/zenodo.8251435. (a) The original stimulus images were overlaid with red lines depicting the resulting optical flow. Three example frames were displayed for each condition. (b) Upon comparing the magnitudes of the optical flows, it was observed that the near-miss stimulus exhibited a slightly larger overall optical flow compared to the hit stimulus. (c) To further analyze the data, we plotted the change in optical flow magnitude at various eccentricities, each shown in different panels. (d) Additionally, we calculated the time-integrated optical flow at different eccentricities. The resulting figure illustrated that the near-miss stimulus generated a greater optical flow in the central visual field when compared to the hit stimulus. Therefore, the collision-sensitive activations in the foveal SC cannot be accounted by a stronger optical flow in the fovea. (e) Similarly, we also obtained the time-integrated luminance change (from the background) for both the hit and near-miss stimuli at different eccentricities. This was accomplis [file pbio.3002375.s002.pdf]

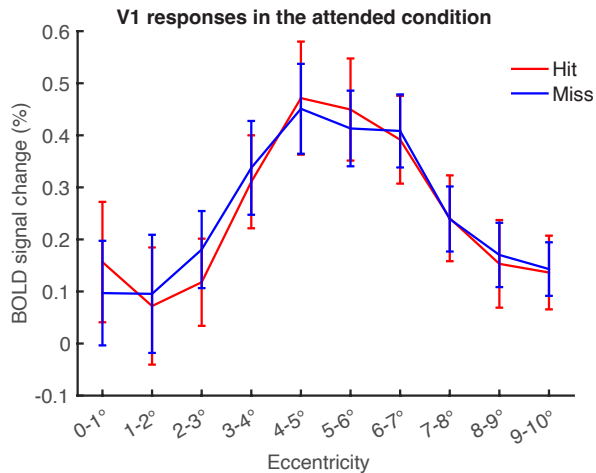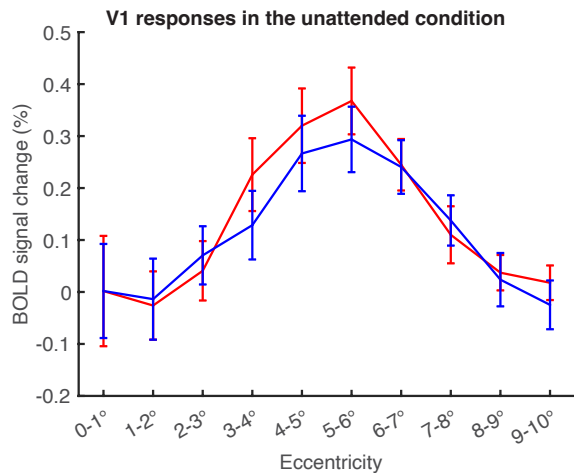

Supplement: S3 Fig — To further support that the stimuli have no systematic bias in retinotopic location, we plotted V1 response profiles as a function of eccentricity. V1 vertices corresponding to the polar angle of the visual stimuli from 0 to 10 degrees of eccentricity were selected based on the HCP retinotopic atlas [68,75]. As shown in the figure below, there was no difference in foveal activations in V1 between the hit and miss stimulus conditions. Therefore, our findings of collision-sensitive activations in the SC cannot be explained by a foveal retinotopic bias to the hit stimulus. (PDF) [file pbio.3002375.s003.pdf]

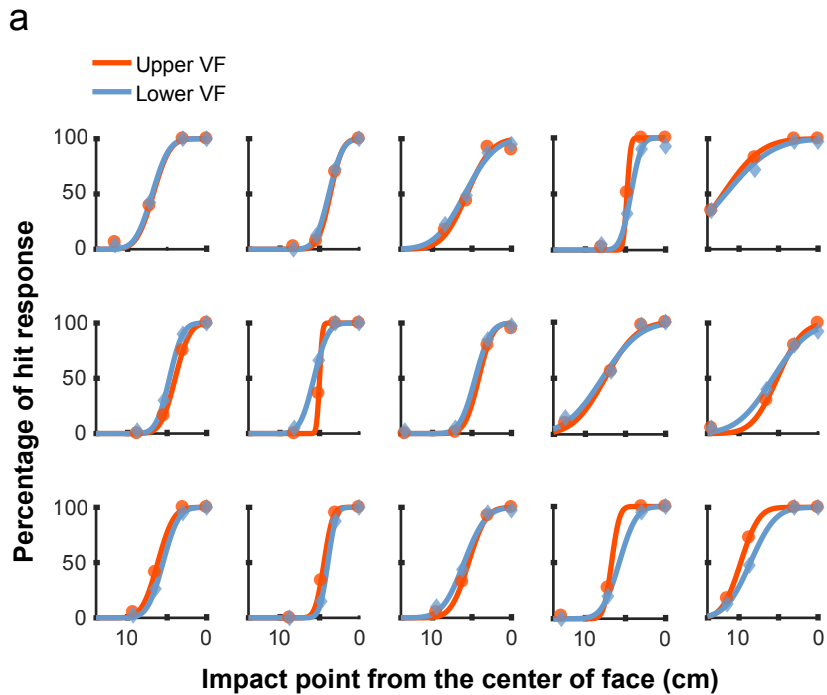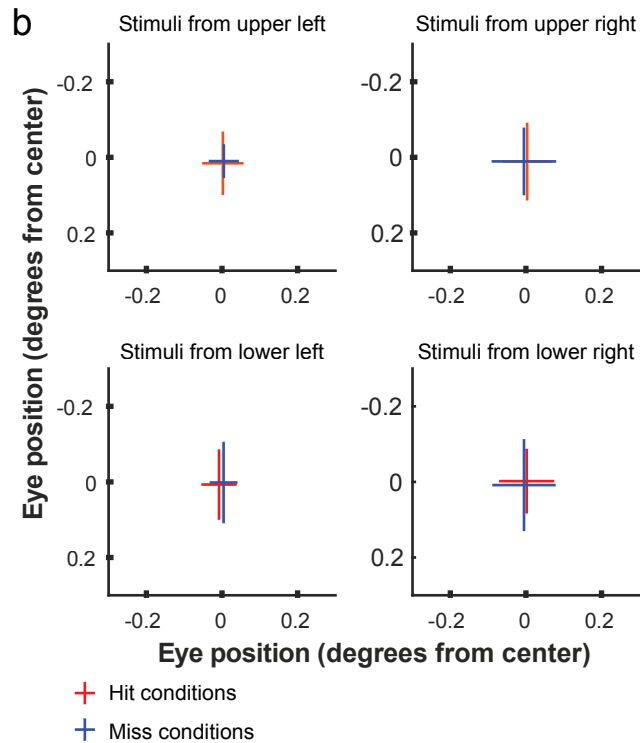

Supplement: S4 Fig — (a) Individual data for the behavioral experiment (experiment 1). The percentage of hit responses to looming stimuli as a function of impact points was fitted with a normal CDF. In experiment 1, the horizontal offset of the would-be impact point for the near miss and far miss conditions was individually adjusted according to the performance of each participant in a preliminary session. The would-be impact point for the near miss and far miss conditions were chosen at approximately 50% and 2% of hit response, respectively. (b) Distribution of eye positions for hit and miss looming stimuli from four quadrants of the visual field. The eye gaze positions from stimulus onset to 1,000 ms after were analyzed. Error bars indicate 3′ standard deviation of the distribution. No significant difference was found between the eye positions to the hit and miss stimuli. (PDF) [file pbio.3002375.s004.pdf]

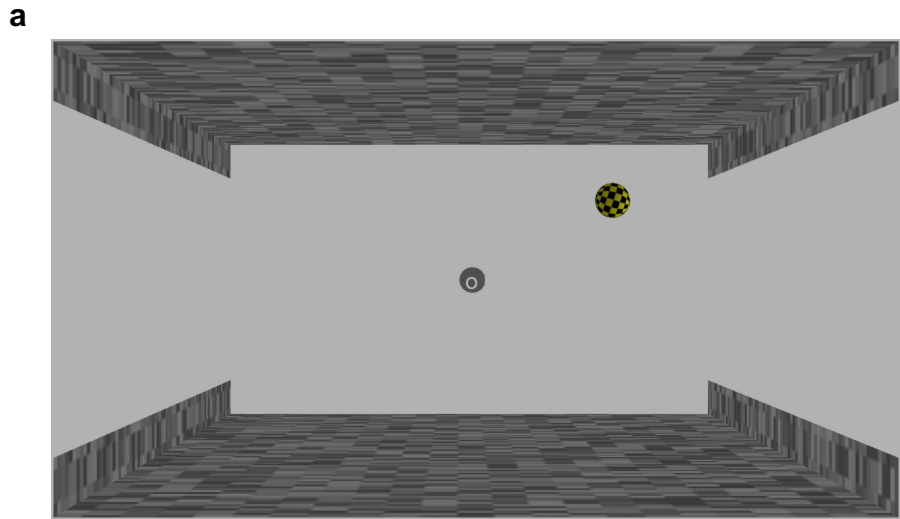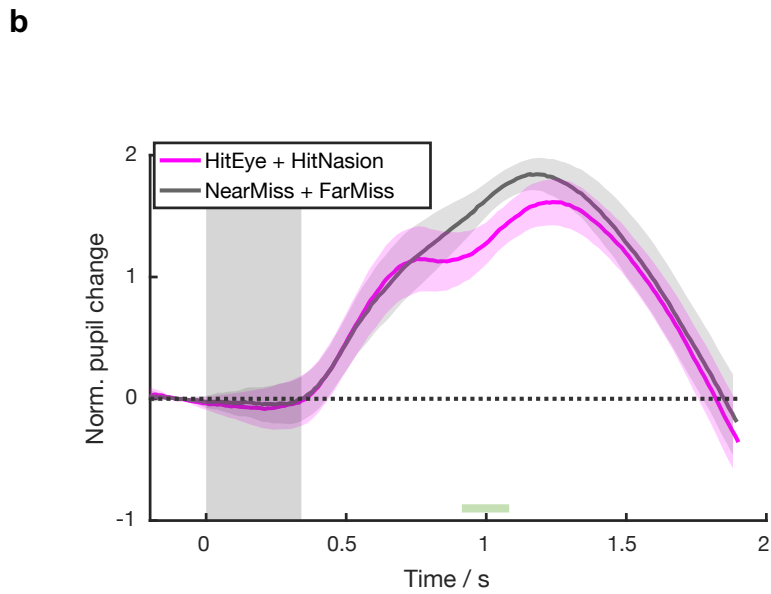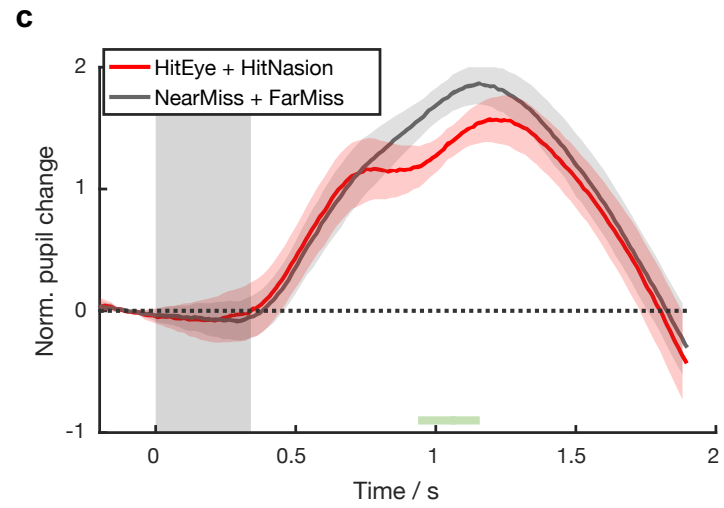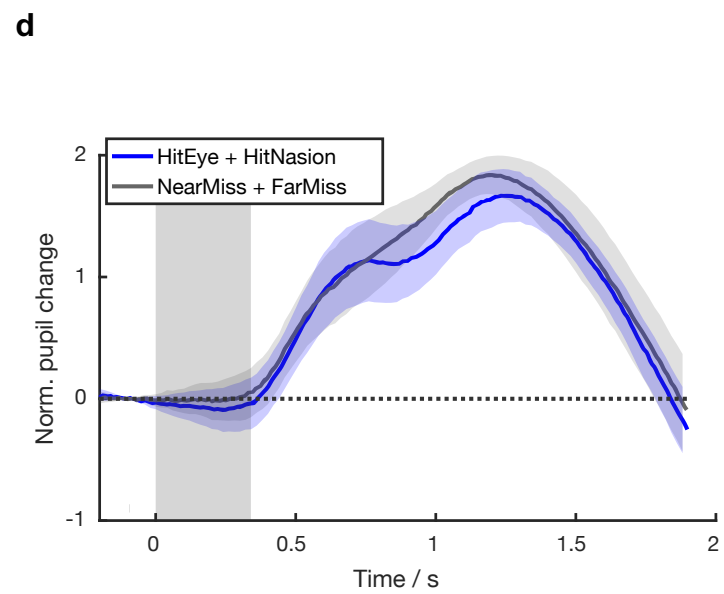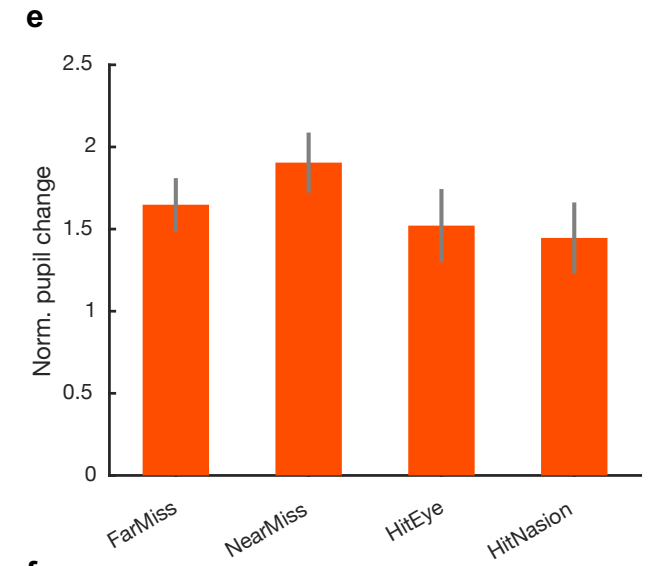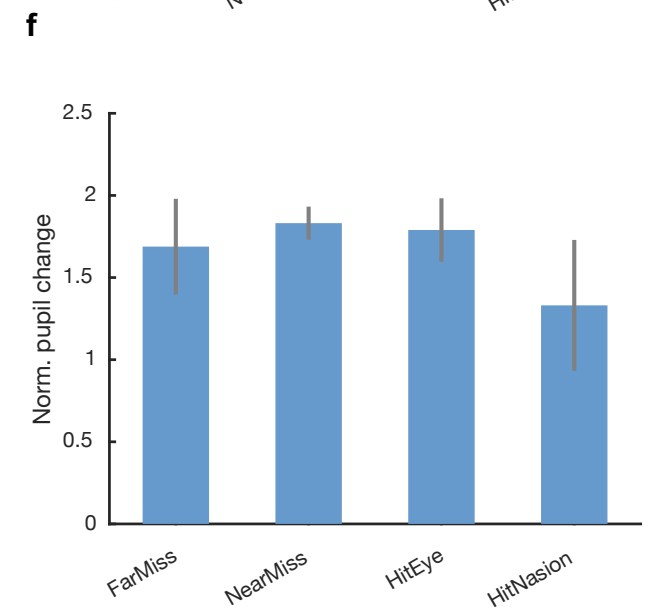

Supplement: S5 Fig — (a) Stimulus diagram was the same as in experiment 1, but with a brighter background than looming stimuli. A total of 10 participants (7 females and 3 males) participated in this experiment. Participants performed a collision detection task, i.e., paying attention to the approaching object. In addition, there was a task-irrelevant rapid presentation of letter streams in the fixation, similar to the fixation change in the unattended condition in experiments 2 and 3 (note in these experiments the fixation change was task relevant). (b, c, d) Time courses of the pupil size change for upper+lower visual field (VF) data, upper VF data only, and lower VF data only, respectively. The light green bar at the bottom indicates the uncorrected significant difference between hit and miss conditions. No significant difference was found after multiple comparison corrections via permutation. (e, f) Bar plots of the pupil size during the looming component (see Fig 1D) for the upper VF only data and lower VF only data, respectively. No significant difference was found between the bars. (PDF) [file pbio.3002375.s005.pdf]

**a**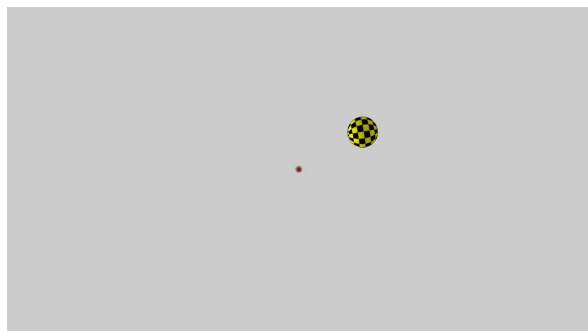**b**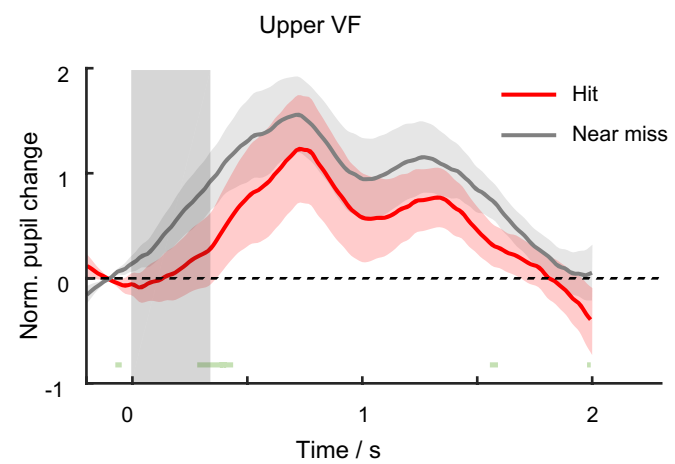**c**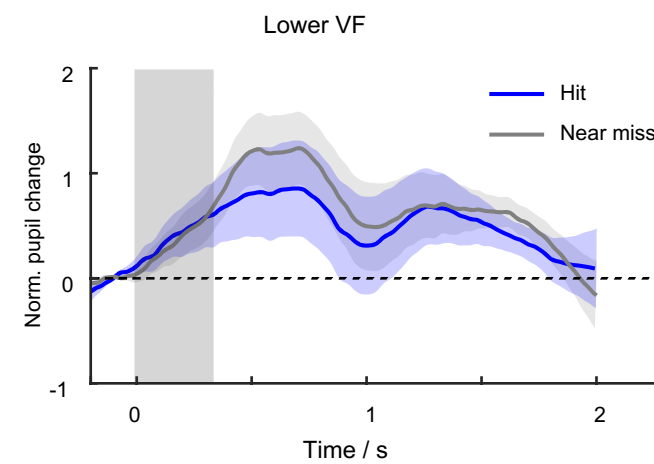

Supplement: S6 Fig — (a) Stimulus diagram was the same as in experiment 2, but with a slightly longer viewing distance (0.85 m). A total of 17 participants (8 females and 9 males) participated in this experiment. Participants were instructed to count the number of color changes of central fixation point. (b, c) The time courses of changes in pupil size for stimuli in the upper (b) and lower (c) visual field are presented. The light green bar at the bottom indicates the uncorrected significant difference between hit and miss conditions. No significant difference was found after multiple comparison corrections via permutation. (PDF) [file pbio.3002375.s006.pdf]

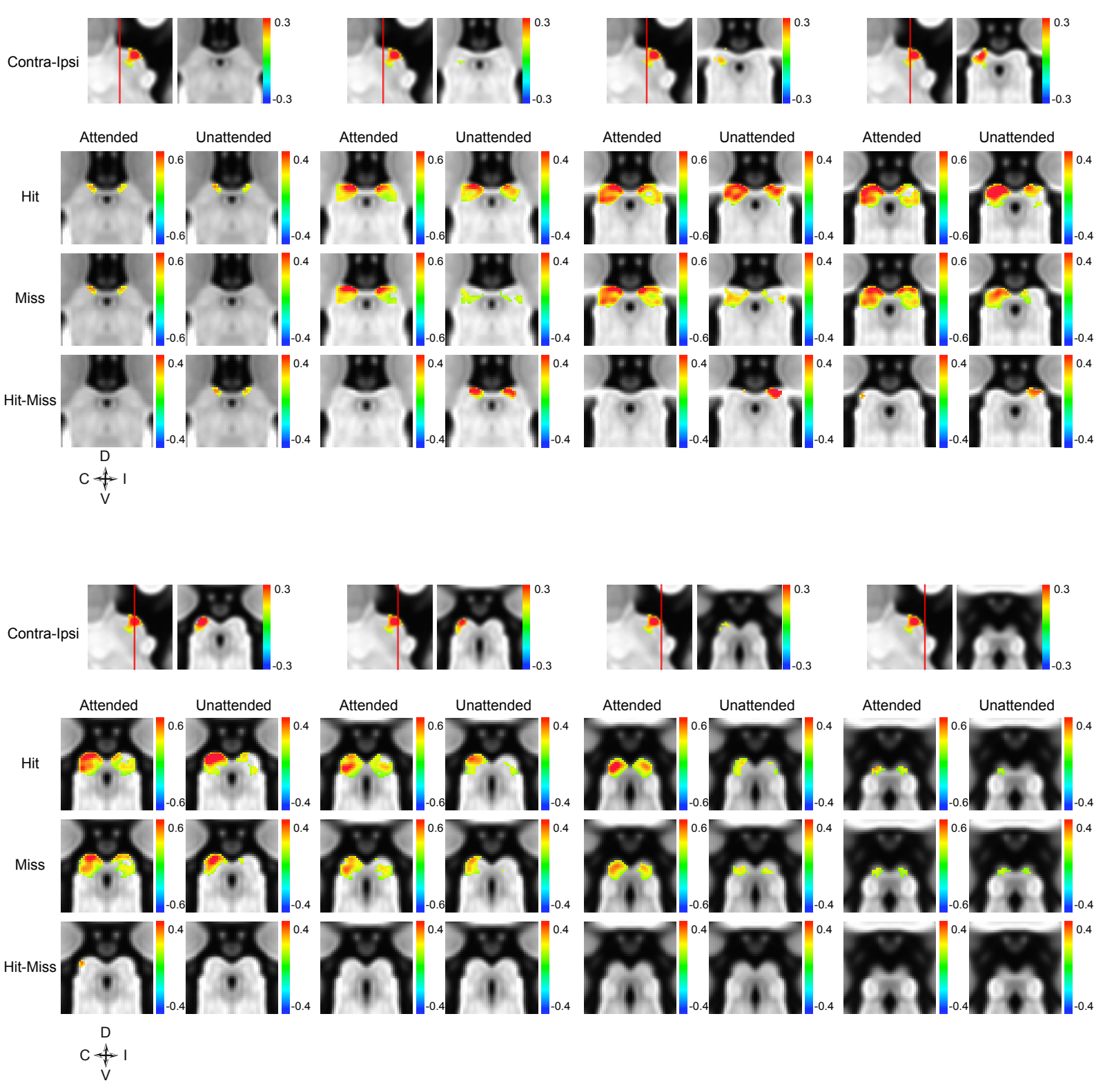

Supplement: S7 Fig — The first row of each panel shows the retinotopic activations with significantly stronger responses to contralateral than to ipsilateral stimuli. Red lines on the sagittal view indicate the location of the coronal slices. The second to the fourth rows show the activation maps for the hit, miss, and hit-miss responses. Maps were thresholded at voxel p < 0.05 uncorrected. (PDF) [file pbio.3002375.s007.pdf]

**SC****VTA****PBGN****LC****Amygdala****LGN****Pulvinar**

Axial

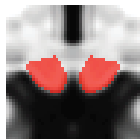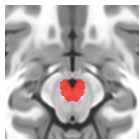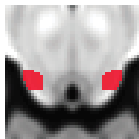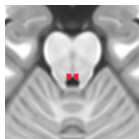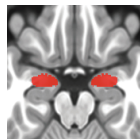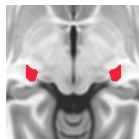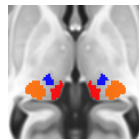

vmPul

vIPul

Coronal

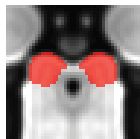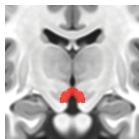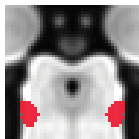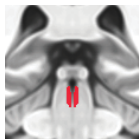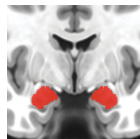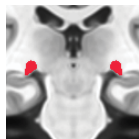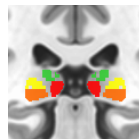

dlPul

dmPul

Sagittal

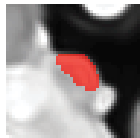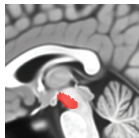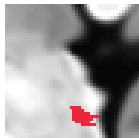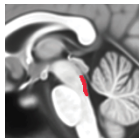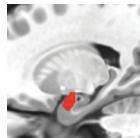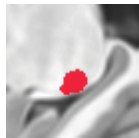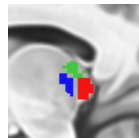

aPul

Supplement: S8 Fig — From left to right are the superior colliculus (SC, 261 μl), ventral tegmental area (VTA, 708 μl), parabigeminal nucleus (PBGN, 90 μl), locus coeruleus (LC, 86 μl), amygdala (1883 μl), lateral geniculate nucleus (LGN, 252 μl), and pulvinar. The pulvinar was parcellated into 5 subdivisions based on the task-coactivation patterns [66], including the ventromedial pulvinar (vmPul, red, 284 μl), ventrolateral pulvinar (vlPul, orange, 359 μl), dorsolateral pulvinar (dlPul, yellow, 246 μl), dorsomedial pulvinar (dmPul, green, 224 μl), and anterior pulvinar (aPul, blue, 215 μl). The pulvinar ROIs used in this study were defined as the intersections of the original ROIs in the left and right hemispheres. (PDF) [file pbio.3002375.s008.pdf]

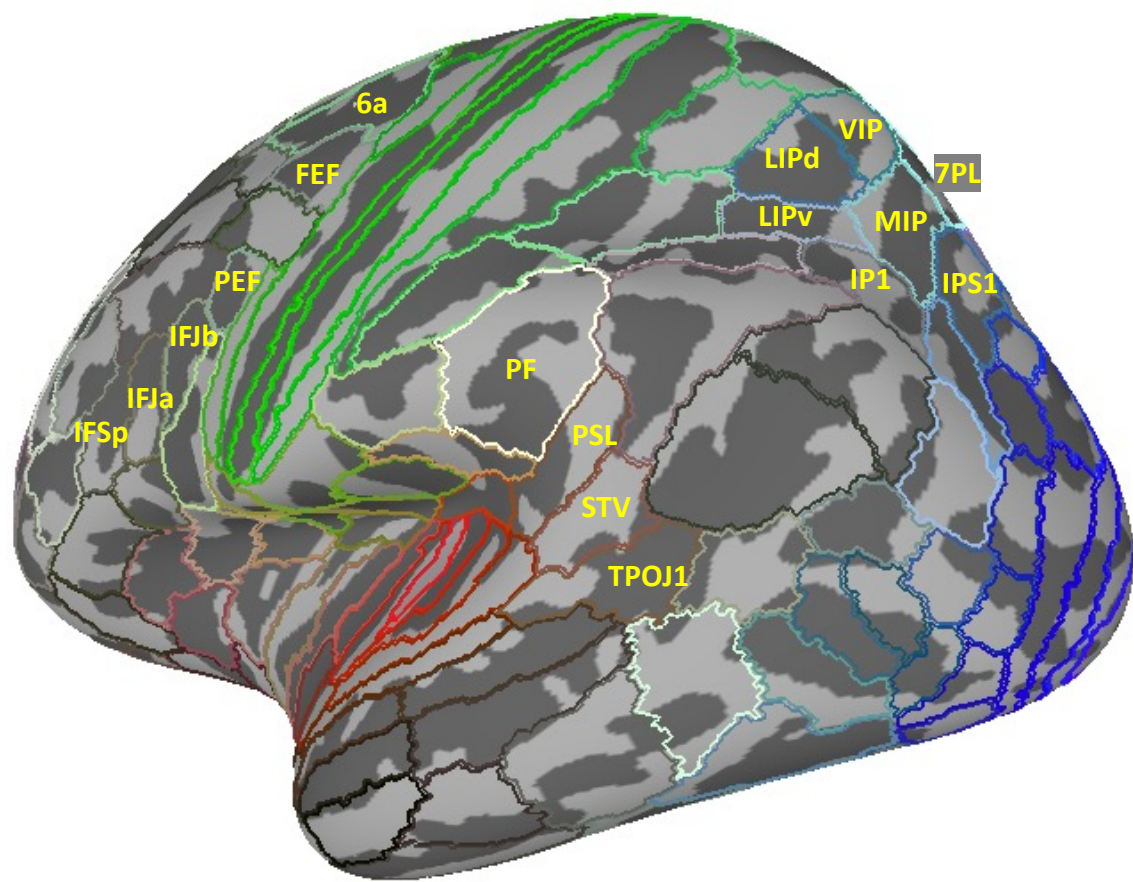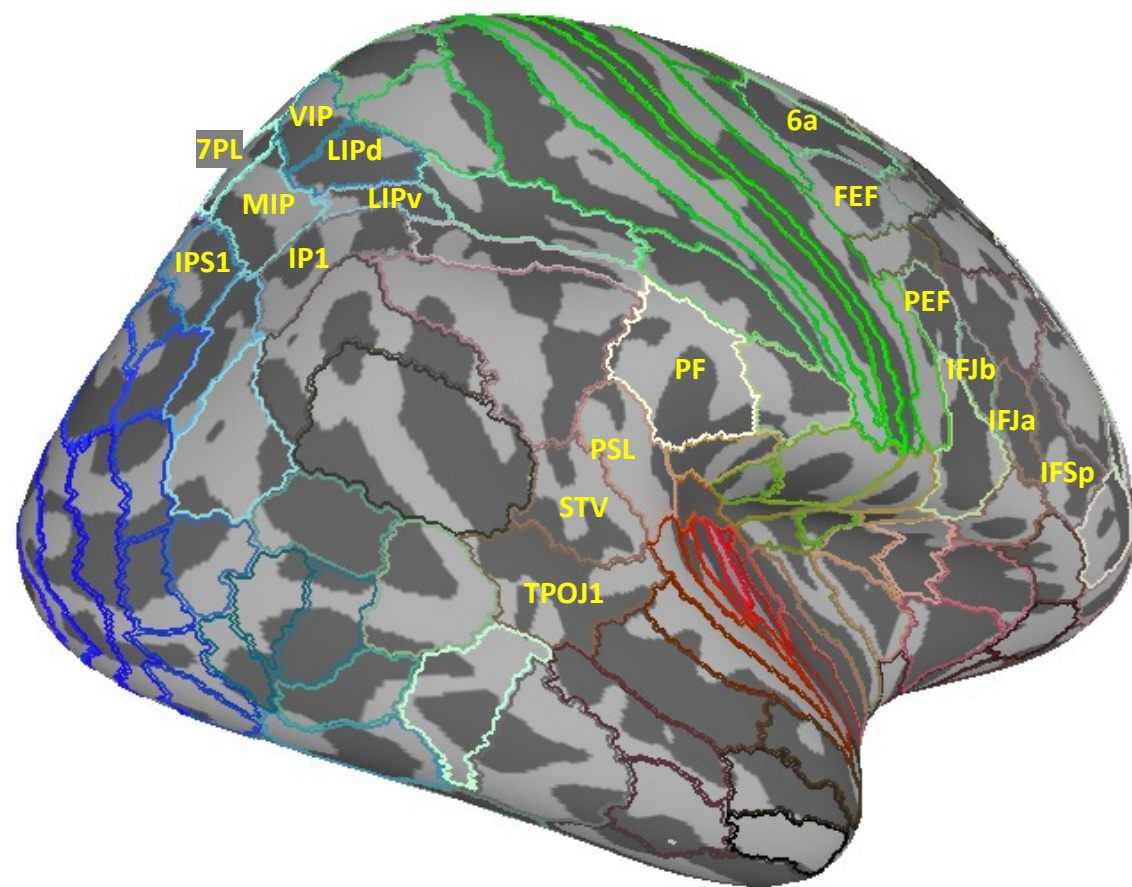

Supplement: S9 Fig — ROIs for dorsal (dAN) and ventral (vAN) attention networks were defined based on anatomical landmarks and HCP-MMP1 atlas. Areas of dAN include IPS/SPL (IPS1, MIP, VIP, LIPv, LIPd, IP1, and 7PL) and SFC (6a and FEF). Areas of vAN include TPJ (TPOJ1, STV, PSL, and PF) and IFC (PEF, IFJb, IFJa, IFSp, and 6r). Yellow annotations indicate selected ROIs in HCP-MMP1 atlas. (PDF) [file pbio.3002375.s009.pdf]

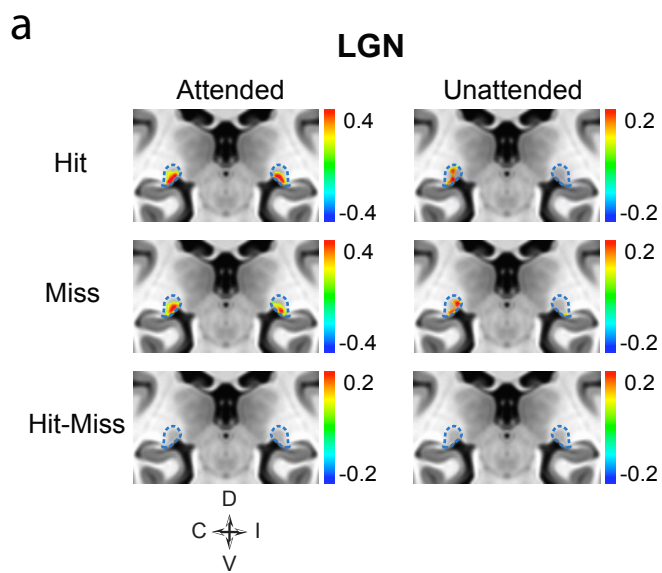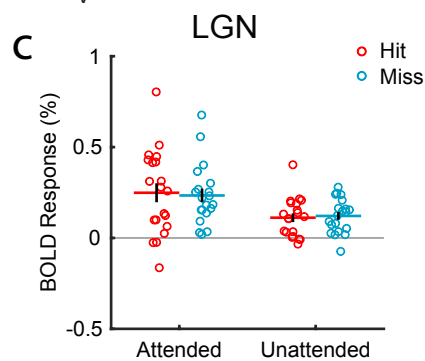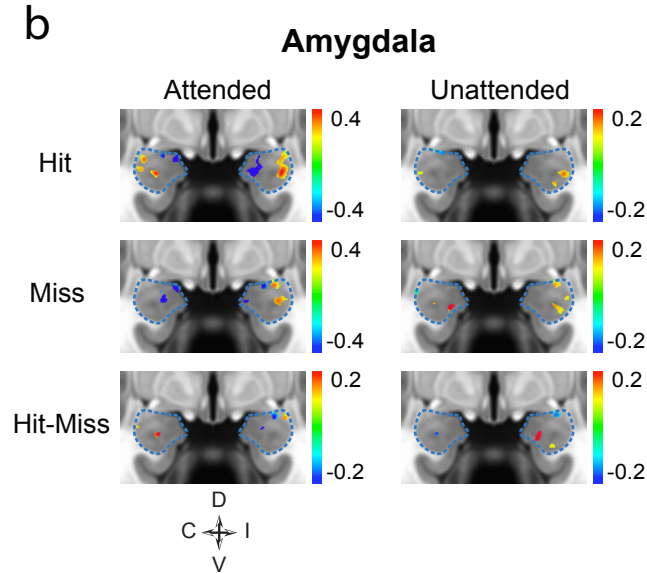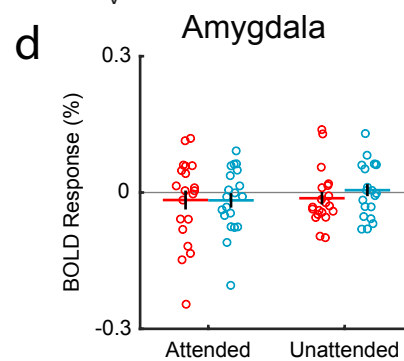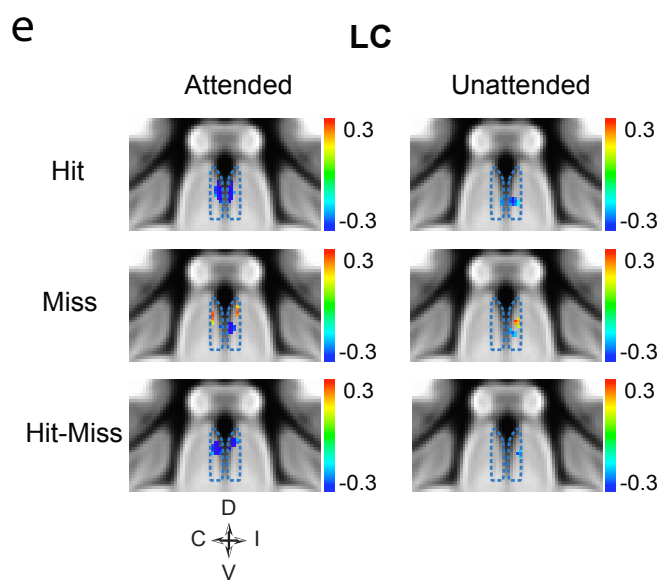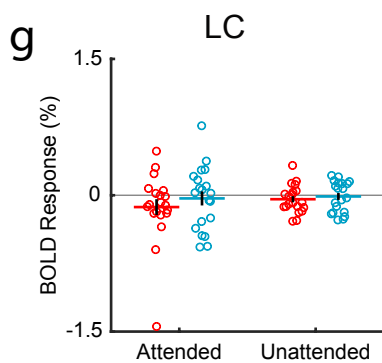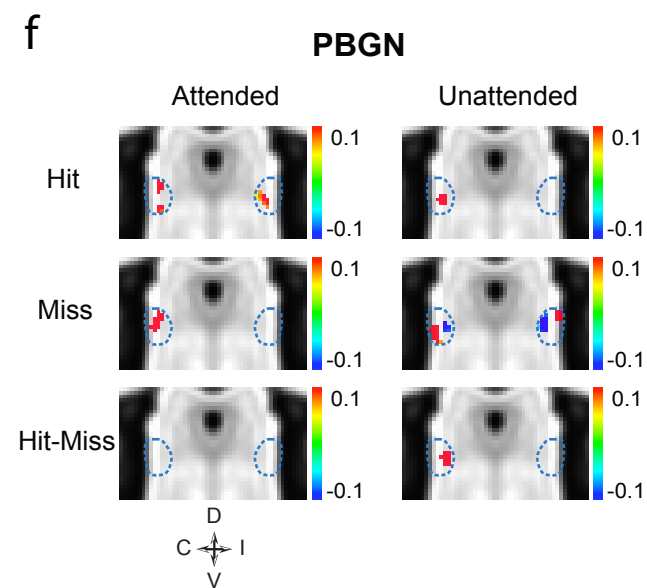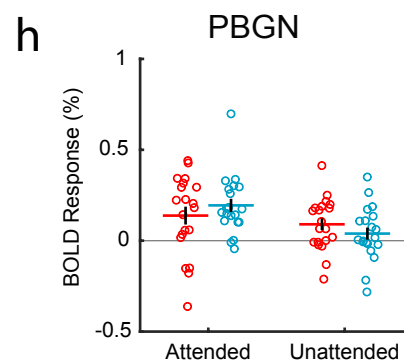

Supplement: S10 Fig — (a, c) LGN. (b, d) Amygdala. (e, g) LC. (f, h) PBGN. Statistical maps were thresholded at p < 0.05 uncorrected. No significant collision-sensitive cluster or ROI-averaged response can be found from these areas. LOSO analysis revealed a significant collision sensitivity in the PBGN in the unattended condition (p < 0.025), but it cannot survive the correction across multiple tests. (PDF) [file pbio.3002375.s010.pdf]

P01

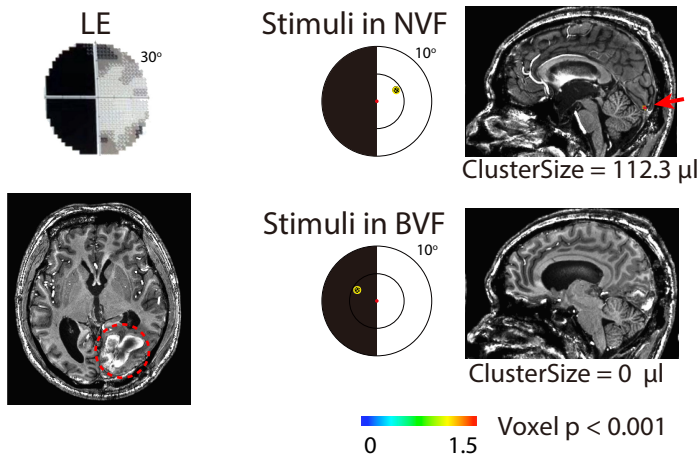

P02

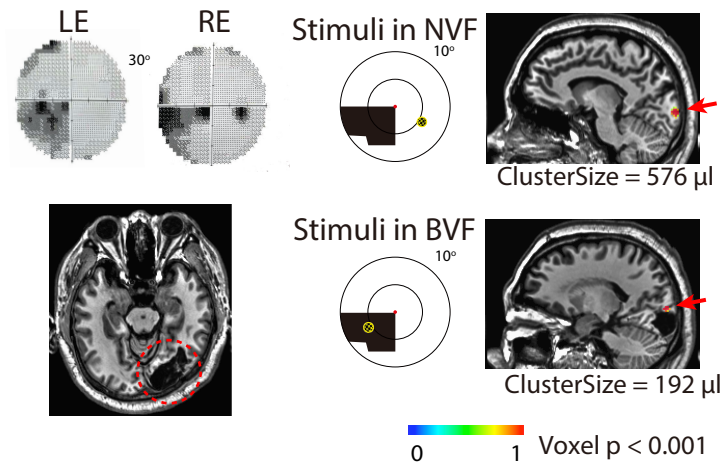

P03

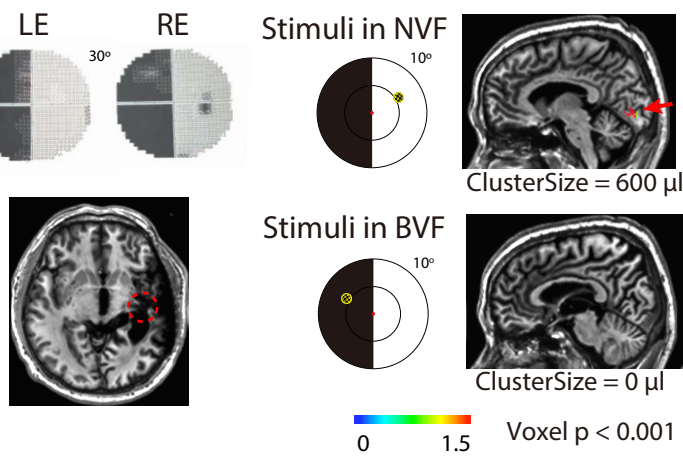

P04

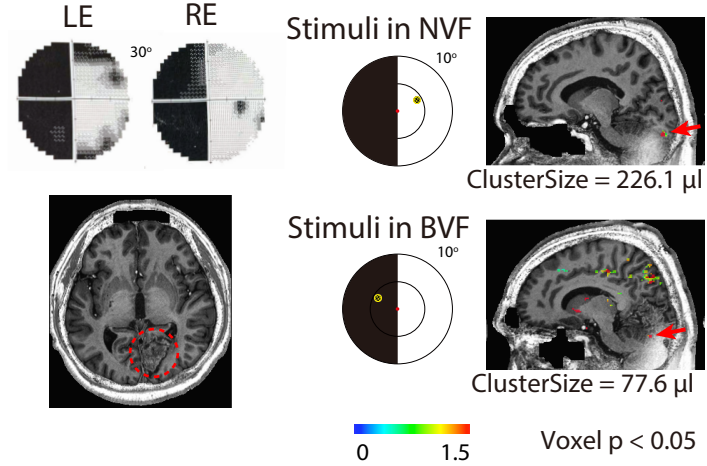

P05

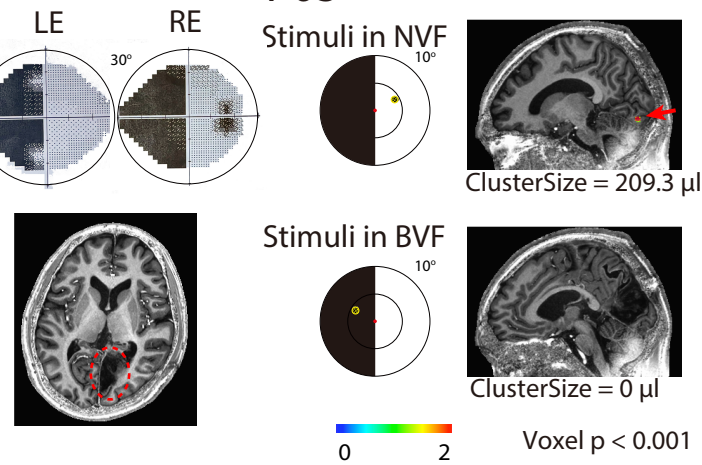

P06

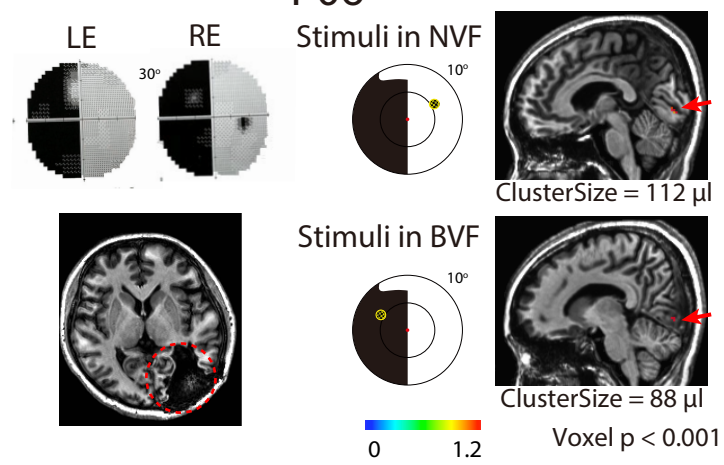

P07

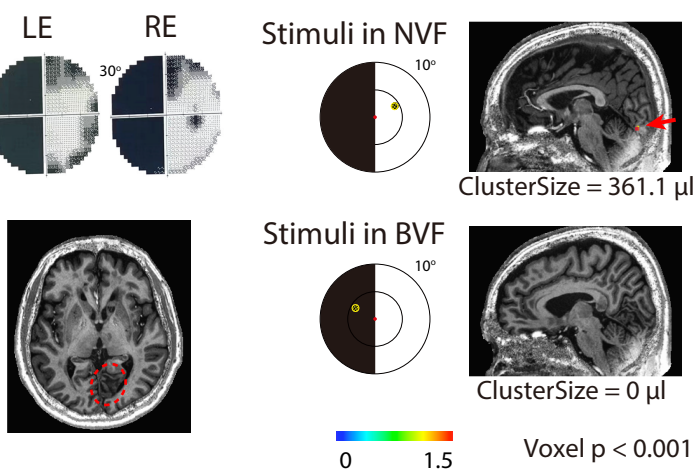

P08

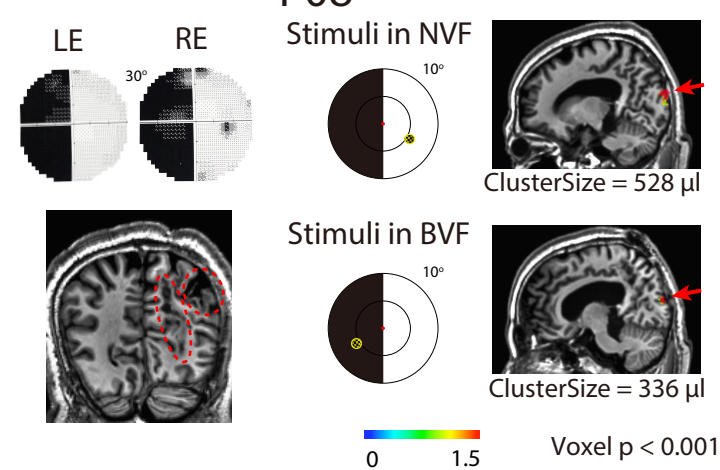

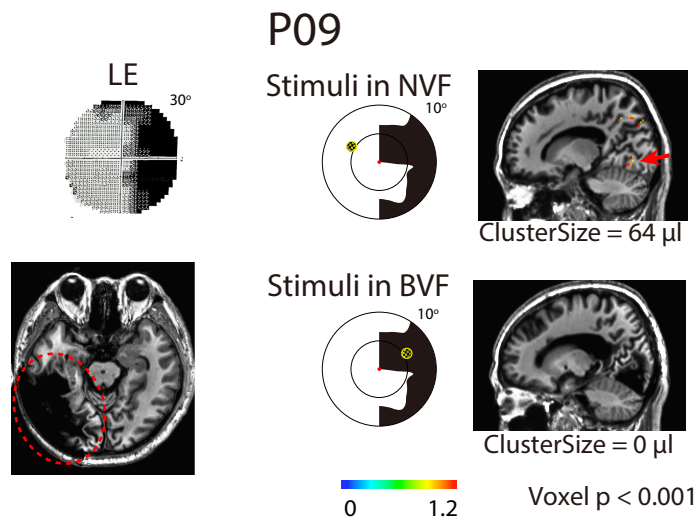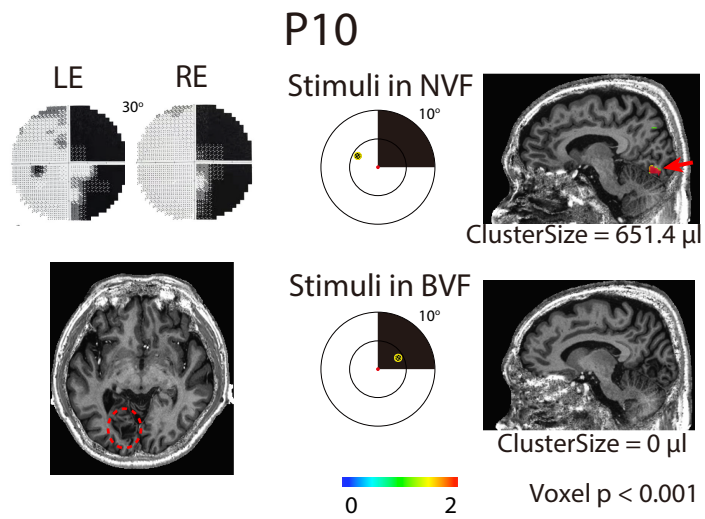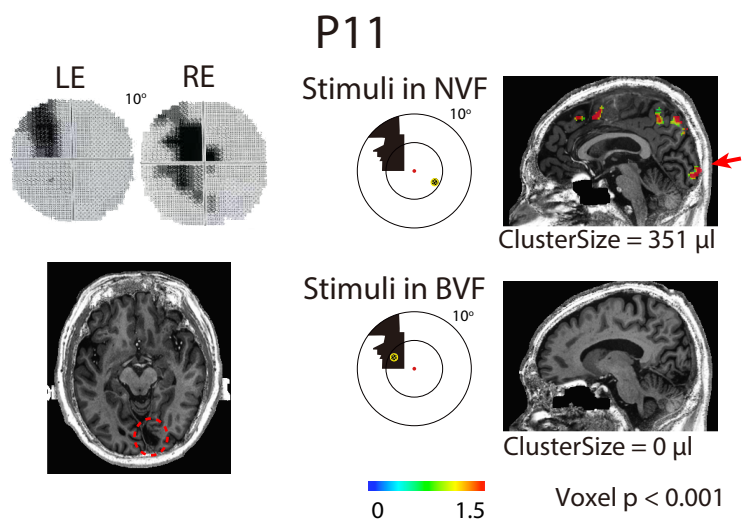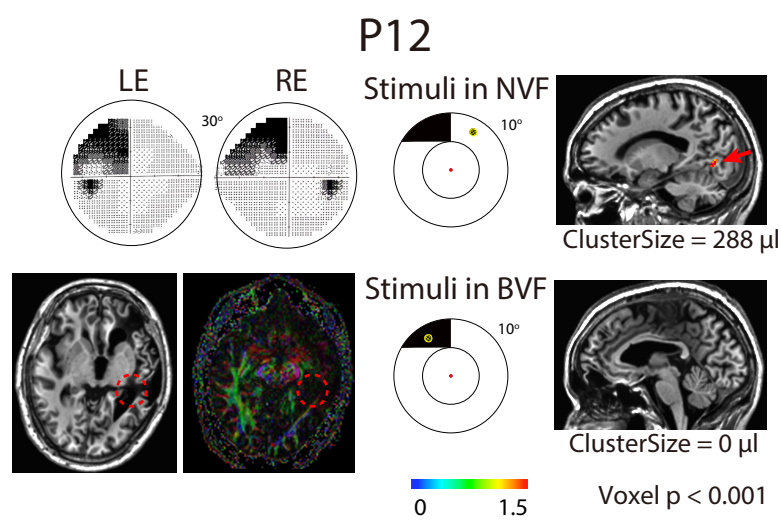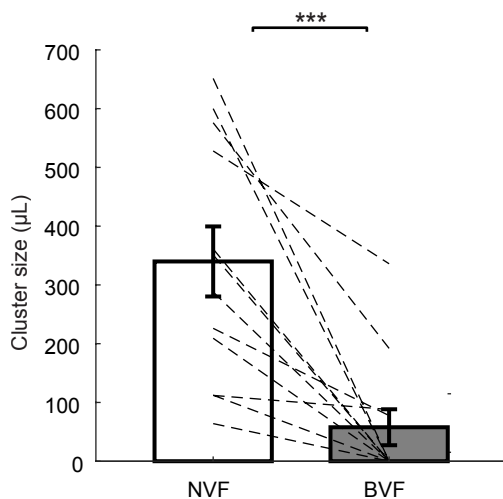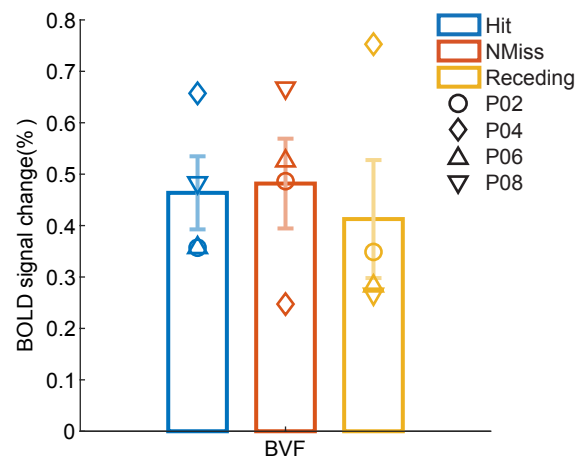

Supplement: S11 Fig — For each patient, the left panels show the Humphrey perimetry of visual field test. In the structural image below, relevant lesioned locations were indicated by red dashed ovals. For P17, both T1w and diffusion tensor images were shown to indicate the lesion of right optic radiation. In the middle panels, the scotoma was depicted schematically within 10 degrees of eccentricity in black color (i.e., relative sensitivity <−20 dB and p < 0.5% compared with normal population), with the yellow sphere indicating the stimulus in the fMRI experiment. The right panels show the occipital activations to stimuli presented to the NVF and BVF (indicated by red arrows). Although clear contralateral V1 activations can be observed to stimuli presented to the NVF, most patients (8/12) showed no significant V1 activation in the lesioned hemisphere to stimulus presented to the BVF (the left bar graph below: dashed line for individual data, *** for p < 0.001). For the 4 patients (P02, P04, P06, and P08) showing weak uncorrected activations in the occipital lobe of the lesioned hemisphere, no significant difference was found in the responses of these voxels to the hit and miss stimuli (the right bar graph below), which cannot explain the collision-sensitive responses in the SC (Fig 6A). (PDF) [file pbio.3002375.s011.pdf]

# LGN

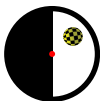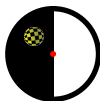

Hit

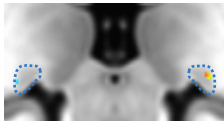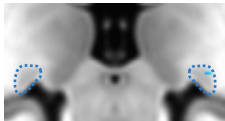

Miss

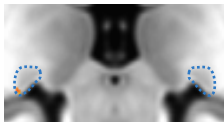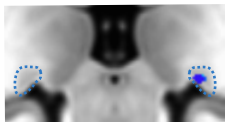

Receding

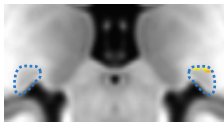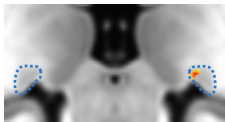

Hit-Miss

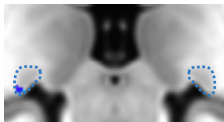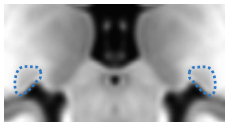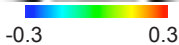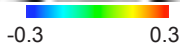

Supplement: S12 Fig — Group-averaged activation maps were thresholded at p < 0.05 uncorrected. Red arrows indicate the location of visually evoked response to receding stimuli in the LGN. Blue dotted lines denote the anatomical boundary of the LGN. No collision sensitivity was found from the ROI-averaged response of the whole LGN, nor from the LOSO analysis. (PDF) [file pbio.3002375.s012.pdf]
