# Supplementary material for: Human subcortical pathways automatically detect collision trajectory without attention and awareness
Source: PLoS Biol. 2024 Jan 18;22(1):e3002375. doi: 10.1371/journal.pbio.3002375 (PMC10795999; doi:10.1371/journal.pbio.3002375)
Supplement: S1 Table — (PDF) [file pbio.3002375.s013.pdf]

PATIENT	SEX	AGE (YEARS)	DAMAGED HEMISPHERE	REASON	AGE AT-LEISION (YEAR)	TIME POST-LESION (MOTHS)	BVF
**P01**	male	38	Right occipital	Tumor Surgery	38	4	Left
**P02**	male	53	Right occipital	Cerebral hemorrhage	52	11	Left
**P03**	male	37	Right	Cerebral hemorrhage	37	1	Left
**P04**	male	37	Right occipital	cerebral infarction	37	1	Left
**P05**	male	25	Right occipital and parietal	Hematoma, Cerebral hemorrhage	25	2	Left
**P06**	female	23	Right	Cerebral hemorrhage	23	5	Left
**P07**	male	63	Right occipital	cerebral infarction	63	1	Left
**P08**	male	42	Right occipital and parietal	Hematoma	42	9	Left
**P19**	male	60	Left occipital	Cerebral hemorrhage	51	104	Right
**P10**	male	57	Left occipital	cerebral infarction	57	2	Right
**P11**	male	64	Right occipital	cerebral infarction	63	16	Left
**P12**	male	47	Right optic radiation	Brain trauma, cerebral hemorrhage	24	280	Left
