# Supplementary material for: Human subcortical pathways automatically detect collision trajectory without attention and awareness
Source: PLoS Biol. 2024 Jan 18;22(1):e3002375. doi: 10.1371/journal.pbio.3002375 (PMC10795999; doi:10.1371/journal.pbio.3002375)
Supplement: S1 Text — (PDF) [file pbio.3002375.s014.pdf]

To compute the optical flow between two images, you must solve this optical flow constraint equation:

$$I_x u + I_y v + I_t = 0$$

- I_x, I_y, and I_t are the spatiotemporal image brightness derivatives.
- u is the horizontal optical flow.
- v is the vertical optical flow.

By assuming that the optical flow is smooth across the entire image, the Horn-Schunck method estimates a velocity field, $[u\ v]^T$, that minimizes this equation:

$$E = \iint (I_x u + I_y v + I_t)^2 dxdy + a \iint \{\left(\frac{\partial u}{\partial x}\right)^2 + \left(\frac{\partial u}{\partial y}\right)^2 + \left(\frac{\partial v}{\partial x}\right)^2 + \left(\frac{\partial v}{\partial y}\right)^2\} dxdy$$

In this equation, $\frac{\partial u}{\partial x}$ and $\frac{\partial u}{\partial y}$are the spatial derivatives of the optical velocity component, u, and α scales the global smoothness term. The Horn-Schunck method minimizes the previous equation to obtain the velocity field, $[u\ v]$, for each pixel in the image. This method is given by the following equations:

$$u_{x,y}^{k+1} = \bar{u}_{x,y}^{k} - \frac{I_x[I_x \bar{u}_{x,y}^{k} + I_y \bar{v}_{x,y}^{k} + I_t]}{a^2 + I_x^2 + I_y^2}$$

$$v_{x,y}^{k+1} = \bar{v}_{x,y}^{k} - \frac{I_y[I_x \bar{u}_{x,y}^{k} + I_y \bar{v}_{x,y}^{k} + I_t]}{a^2 + I_x^2 + I_y^2}$$

In these equations, $[u_{x,y}^k\ v_{x,y}^k]$ is the velocity estimate for the pixel at (x, y), and $[\bar{u}_{x,y}^k\ \bar{v}_{x,y}^k]$ is the neighborhood average of $[u_{x,y}^k\ v_{x,y}^k]$. For $k = 0$, the initial velocity is 0.
To solve u and v using the Horn-Schunck method:

1. Compute I_x and I_y by using the Sobel convolution kernel, $[-1\ -2\ -1;\ 0\ 0\ 0;\ 1\ 2\ 1]$, and its transposed form for each pixel in the first image.
2. Compute I_t between images 1 and 2 using the $[-1\ 1]$ kernel.
3. Assume the previous velocity to be 0, and compute the average velocity for each pixel using $[0\ 1\ 0;\ 1\ 0\ 1;\ 0\ 1\ 0]$ as a convolution kernel.
4. Iteratively solve for u and v.
