# Supplementary material for: Human subcortical pathways automatically detect collision trajectory without attention and awareness
Source: PLoS Biol. 2024 Jan 18;22(1):e3002375. doi: 10.1371/journal.pbio.3002375 (PMC10795999; doi:10.1371/journal.pbio.3002375)
Supplement: Attachment — Submitted filename: Response to the reviewers.docx [file pbio.3002375.s015.docx]

Reviewers' comments:
Reviewer #1 (Remarks to the Author):

The authors have sufficiently addressed all my previous comments and concerns.
Re: Thank you for your valuable comments and suggestions. We are glad that all concerns from you and reviewer #2 have been fully addressed.

Reviewer #3 (Remarks to the Author):

Thank you for the response to questions. However, I still have concerns that some of them have not been addressed/ or changes have just created new problems. I’ll focus on the non-patient data and the major issues. 

Issues still apparent with defining ROIS

The split half-runs approached used does not appear to have been done correctly – from the description provided. They still appear to be using all the data to define the ROI and all the data to analysis. There would surely still be dependence here. The Split-Half-Runs approach is not this… it’s using half the data as a functional ROI and half the data for analysis, once. This is an independent functional localizer approach. What are the results if the authors report “Data from either the odd or even half of runs were used as an independent localizer to define the retinotopic ROIs of visual cortex, from which ROI-averaged responses were extracted for the other half of data” without the “Subsequently, the same analysis was repeated by switching the localizer and experimental runs, and the two results were averaged together”. If this was significant I’d be more convinced of the result. 
Split half runs approach papers - Vul et al. 2009 <https://doi.org/10.1111/j.1745-6924.2009.01125.x/> Poldrack et al. 2009 10.1093/scan/nsp011
Line 696 “The collision sensitive voxels for each ROI were defined by the LORO approach described from above” 
This approach seems to have the same issue as the split half runs approach?

Re: We would like to clarify that the split-half-run and leave-one-run-out (LORO) analysis in this study is indeed the K-fold cross-validation approach. It is a widely used method in machine learning and statistical analysis to avoid the problem of overfitting (or double dipping). During analysis, our data were split into K fold (K = 2 and 4 in split-half-run and LORO analysis, respectively), K-1 fold of data were used to select ROIs and the remaining fold was used as the test data. The procedure was repeated K times, with each fold severed as the test data once. The results from all iterations were then averaged together as the final estimate.

Pereira et al. (2009) provided a clear explanation of K-fold cross-validation in fMRI data analysis. On page S203 of the main text:

*“there is a procedure that allows us to have our cake and eat (almost) all of it too. The procedure is called cross-validation. In its most extreme variant, cross-validation follows these steps:*

*• leave one example out, train on the remaining ones, make a prediction for this example*

*• repeat for each example in turn*

*• compute the accuracy of the predictions made for all the examples.*

*…*

*In practice, leaving each example out can be computationally expensive because instead of a single classifier we are training as many classifiers as there are examples. One compromise is a procedure called a k-fold cross-validation, where k is the number of parts into which the dataset is divided; common choices are k=10 or k=5, corresponding to leaving out 10% or 20% of the examples on each fold.”*

This method was suggested to avoid the problem of double-dipping in the seminal paper by Kriegeskorte (2009), on page 2 of supplemental discussion:

*“Crossvalidation allows us, in a sense, to have our cake and eat it too: by splitting off small independent subsets of the data repeatedly, we can take advantage of the benefits of a split-data analysis, and* ***still use almost all data for fitting and all data testing****. The price of this is added computational demands. The implementation of a correct crossvalidation scheme requires some care as each split-off subset needs to be independent of the remainder of the data. Importantly, the selection process must be performed independently for each crossvalidation fold.”*

It was also recommended by Vul et al., 2009, on page 283:

*“For an even more data-efficient (but computationally intensive) independent validation technique, variants of the ‘‘k-fold method’’ can also be used (Brieman & Spector, 1992).”*

References:

*Pereira, F., Mitchell, T., & Botvinick, M. (2009). Machine learning classifiers and fMRI: a tutorial overview. NeuroImage, 45(1 Suppl), 199–209.* [*https://doi.org/10.1016/j.neuroimage.2008.11.007*](https://doi.org/10.1016/j.neuroimage.2008.11.007) *(****1838 citations from google scholar)***

*Kriegeskorte, N., Simmons, W. K., Bellgowan, P. S., & Baker, C. I. (2009). Circular analysis in systems neuroscience: the dangers of double dipping. Nat Neurosci, 12(5), 535–540.* [*https://doi.org/10.1038/nn.2303*](https://doi.org/10.1038/nn.2303) ***(2671 citations from google scholar)***

*Vul, E., Harris, C., Winkielman, P., & Pashler, H. (2009). Puzzlingly High Correlations in fMRI Studies of Emotion, Personality, and Social Cognition1. Perspectives on Psychological Science, 4(3), 274–290.* [*https://doi.org/10.1111/j.1745-6924.2009.01125.x*](https://doi.org/10.1111/j.1745-6924.2009.01125.x) *(****1742 citations from google scholar)***

To further demonstrate that our split-half-run (or 2-fold) cross-validation approach does not produce bias to the results of ROI-averaged responses, we ran a simulation analysis. Two runs of random data (no effect) were generated in an ROI with 1000 voxels for 100 subjects. In the double-dipping analysis, we selected the most responsive 5% voxels from each run and tested the effect from these voxels in the same run, and the results from run1 and run2 were averaged. In the 2-fold cross-validation analysis, we defined the most responsive voxels from run1 and test the averaged response of these voxels in run2, and vice versa. The results from both iterations were averaged as the final estimate. The violin plot in the figure below shows the mean and distribution of the ROI-averaged responses from both analyses. The 2-fold cross-validation analysis clearly demonstrates no bias in mean responses.

I also cannot understand why the switch of approach? Why not use split half runs OR LORO approach for all analysis? Why switch? 
Re: The switch in approach was motivated by the different purposes served by each method. We utilized the split-half-run approach to examine whether the retinotopic responses in visual cortical areas are stronger for hit compared to near-miss stimuli. The reason we chose this approach here is its computational simplicity and that data from half (two) runs are sufficient to reliably localize visually responsive voxels.

On the other hand, for the correlation and functional connectivity analysis of collision sensitive activations among brain regions, we used the LORO approach. This method provides more training data (three runs), enabling accurate localization of collision sensitive voxels, which is crucial for this analysis. The higher collision sensitivity with the LORO approach is depicted in the figure below.

In the revised manuscript, we used the LORO approach for the analyses of both retinotopic responses in visual cortical areas, and the correlation/connectivity analysis of collision sensitivity among brain regions. The LORO results of retinotopic responses in the early visual areas (the figure below) showed weaker overall collision sensitivity compared to the results of split-half-run approach, this is due to a larger retinotopic ROI defined by 3 (K-1) runs of data compared to 2 (half) runs (e.g. 114.8±16.2 compared to 66.6±9.2 voxels in V1, *p* < 0.05 uncorrected).

Also there are still some sections of the paper where they still are not using corrective techniques.

Re: Statistical details about multiple corrections across subcortical ROIs were provided in the previous response letter. They were now included in the main text (line 279-282 in results, line 859-871 in methods). The *p* values of SC-VTA and SC-vmPul correlations cannot survive multiple corrections of the correlation matrix by permutation test, they were clearly labeled as uncorrected in the main text. However, since we have a strong prior to test the relationship of SC with its downstream subcortical nuclei and cortical areas, the SC-VTA and SC-vmPul correlations were indeed significant after Holm correction among these connections (*p* = 0.036 and 0.030, respectively). Importantly, the beta-series and SEM path analysis demonstrated highly significant SC-VTA and SC-vmPul connections (all *p* < 0.001 after correction).

Why not use appropriate split half runs approach in this section of the paper? “The frontoparietal ROIs were manually defined on the cortical surface based on group-level activation to all looming stimuli (Supplementary Fig. 8), they were only used for the correlation and effective connectivity analysis.” 
Re: Supplementary Fig.8 shows the manually defined study-specific atlas (or surface mask) for the frontoparietal areas, based on group-level activations of all stimuli. In the correlation and connectivity analysis, collision-sensitive voxels in the frontoparietal areas were defined by the LORO approach at the individual level. LORO approach was used for all brain areas in this analysis. We used LORO rather than split-half-run approach in this analysis because LORO provides more training data to accurately locate collision sensitive voxels, which is critical to study the correlation and connectivity of collision sensitive activity among brain regions.

To avoid any possible issue with circular testing, we now defined the surface ROIs for the dorsal and ventral frontoparietal attention networks based on anatomical landmarks and the HCP-MMP1 atlas (Supplementary Fig.8, also shown as the figure below, yellow annotations indicate selected frontoparietal ROIs) (Glasser et al., 2016).

Reference:

*Glasser, M. F., Coalson, T. S., Robinson, E. C., Hacker, C. D., Harwell, J., Yacoub, E., Ugurbil, K., Andersson, J., Beckmann, C. F., Jenkinson, M., Smith, S. M., & Van Essen, D. C. (2016). A multi-modal parcellation of human cerebral cortex. Nature, 536(7615), 171–178. https://doi.org/10.1038/nature18933*

Unclear results

This result still isn’t clear to me….”In the extrafoveal SC (Fig. 2f), there was a significant main effect of trajectory (F(1,19) = 6.75, p = 0.018, ߟ௣ ଶ 218 = 0.262). Post-hoc t-tests of the difference between hit and miss responses were not significant in both attended and unattended conditions”. This is a 2x2 design (attention (2 levels) x hit (2 levels)), so why are post hocs being run? And how can they be non-significant if the main effect is significant with only 2 levels? Also figure 2f still appears to have no significant markers on it. I brought up this question in my last review. 
Re: It is indeed possible to have a significant main effect in a 2x2 ANOVA without significant post-hoc tests. The main effect in a 2x2 ANOVA represents the overall effect of one independent variable (Factor A, i.e. trajectory) while ignoring the other independent variable (Factor B, i.e. attention). It tests whether there is a significant difference between the levels of Factor A (hit and near-miss), regardless of the levels of Factor B (attended and unattended). On the other hand, post-hoc tests are conducted to examine specific pairwise comparisons between groups or conditions after a significant main effect is found. These tests help determine which specific groups differ significantly from each other. In the extrafoveal SC, the main effect of trajectory is significant, but the post hoc tests between hit and near-miss responses in attended and unattended conditions are not. This could be due to smaller effect sizes that affect the power of the post-hoc tests.

We now added a significance marker to indicate the main effect of trajectory in Figure 2f. The statistical results have been clearly described in the main text.

*Line 218-221: “In the extrafoveal SC (Fig. 2f), there was a significant main effect of trajectory (F(1,19) = 6.75, p = 0.018,* $\eta_{p}^{2}$ *= 0.262), but no interaction between attention and trajectory (p = 0.943). This finding suggests collision sensitivity in the extrafoveal SC independent with the attentional state of the observer.”*

I fundamentally disagree with discussing p=0.091 as “marginally significant”, especially with so many analysis / inconsistent approaches to correction. The authors disagree and are still discussing these results as if they are significant – I will leave that decision with the Editor. 
Re: Following your suggestion, we now removed the term “marginally significant”. However, we need to clarify that this *p* value denotes the significance of a collision-sensitive cluster in the ventrolateral pulvinar (vlPul) in the attended condition. Since the conclusion of the study primarily focused on the unattended condition, the insignificance of cluster *p* in the attended condition does not affect our main conclusion. Moreover, there were also additional evidence supporting collision sensitivity in vlPul in the attended condition, both anatomical ROI-averaged responses and LOSO cross-validation result showed significant collision sensitivity (*p* = 0.013 and 0.016, respectively).

Confounding factors

The authors have now run a Horn-Schunck method to compute flow. It would be good if they reported considerable more detail on how they computed and implemented this and include code as supplementary would be wise. The trajectories in Figure S2 doesn’t quite look as I’d expect, in figure S1 the balls all seem to move on linear trajectories, but the trajectory from the 3 frame provided seems to look curvilinear? Perhaps this is just perception on screen but it would be good to state which frames you’re showing and more detail on this analysis.

Re: The Horn-Schunck method is utilized to determine the optical flow by analyzing the displacement of pixels between frames, assuming that the brightness of a pixel remains constant during its motion. In our analysis, we captured the image in each frame during the visual stimulation. Subsequently, we employed the MATLAB Optical Flow block (the mathematical algorithm is given at the end the letter^[1]^) from the Computer Vision Toolbox to estimate the optical flow vector for each pixel between two consecutive frames. The direction of pixel motion was determined by the horizontal and vertical components of the vector, while the speed or magnitude was calculated as the square of the vector's modulus. Finally, we computed the overall flow magnitude by summing the magnitudes of all pixels. The scripts for this analysis have been uploaded to <https://doi.org/10.5281/zenodo.8251435>. Following your suggestion, the computation details for optical flow were now included in the legend of supplementary fig.2.

The number of selected frames (#4, #12, #20 from stimulus onset) were now denoted in supplementary fig. 2b.

Indeed, the curvilinear path observed in the three frames was a result of the visual perception on the screen. We have illustrated this in a figure where the objects have been distorted in their proportions to express the idea clearer. Within this figure, it can be observed that $l_{1}=l_{2}$, whereas $l_{1}^{'}<l_{2}^{'}$.

Further, there is still a large confound. The result are only significant for foveal SC, so although the authors have worked out overall motion energy, there would still be more motion energy in the center of the screen for the hit response (i.e. where the foveal SC is sensitive to?). The hit stimuli enters central fovea and the miss stimuli does not? So this could just be a stimuli difference and not a looming difference? This issue would only be solved with some SC retinotopic mapping (Keith Schneider has done some great work on this). 
Re: The collision-sensitive cluster in the foveal SC was not in the retinotopic region corresponding to the Hit stimulus. The figure below shows the retinotopic activation to the Hit stimulus (contralateral – ipsilateral, *p* < 0.001 uncorrected) in the contralateral SC, and the collision-sensitive clusters (Hit-Miss, *p* < 0.05 uncorrected) in bilateral SCs. Blue dotted lines indicate the ROIs for the foveal and extrafoveal SCs. Collision-sensitive activations are clearly located outside the retinotopic regions of the contralateral SC, and also in the non-retinotopic ipsilateral SC. Thus, collision-sensitive activation outside the retinotopic region of the hit stimulus is irrelevant to the retinotopic bias.

There might be a misunderstanding about the looming stimuli. Since we aligned the center of mass for the hit and near-miss trajectories projected on screen, the near-miss stimulus is actually located closer to the fixation for most of the frames (18 out of 20 frames, from frame#1 to frame #18), while the hit stimulus is closer to fixation only at the end of presentation (2 out of 20 frames, from frame#19 to frame#20). To evaluate the overall magnitude of optical flow during stimulus presentation, we calculated the time-integrated optical flow at different eccentricities. As depicted by the figure below, the spatial distributions of optical flow for the two looming trajectories are very close. The near-miss stimulus even shows more optical flow at the lower eccentricity compared to the hit stimulus. Therefore, collision-sensitive activation in the foveal SC is unlikely due to the stimulus difference in optical flow.

To further support that the stimuli have no systematic bias in retinotopic location, we plotted V1 response profiles as a function of eccentricity. V1 vertices corresponding to the polar angle of the visual stimuli from 0 to 10 degrees of eccentricity were selected based on the HCP retinotopic atlas (Benson et.al, 2014, 2018). As shown in the figure below, there was no difference in foveal activations in V1 between the hit and miss stimulus conditions. Therefore, our findings of collision-sensitive activations in the SC cannot be explained by a foveal retinotopic bias to the hit stimulus.

Line 596: “The luminance change and overall magnitude of optical flows (Supplementary Fig. 2) 597 were similar between the two trajectory conditions. The stimulus on the screen expanded from 598 0.4 to 4.5 degrees of visual angle in 330 ms, at an eccentricity of about 5 degrees (angle with 599 vertical meridian is 60º).” 

Figure 2 does not even mention luminance?
“The difference in overall luminance changes between the hit and miss conditions should be minimal, since we aligned the center of mass for the retinal projections of different looming trajectories” 

The authors have not addressed or measured luminance in difference parts of the screen in their response and have just said it “should be minimal”. This is vague. It would be perfectly feasible to measure this retrospectively with a luminance meter? 
The center of mass isn’t really the issue, it’s the large increase in foveal luminance for the hit condition, which highly likely driving either some or part of the foveal SC. Further, the result is only significant for unattended (Figure 2e). This is the condition where participant’s eye-movement are most likely to be stably fixated, because of the secondary task. In the attended condition there is a likely to be drift towards the stimuli (reducing condition difference in luminance and also making SC response higher overall compared to unattended – which is the case) – and there’s no in scanner data to prove this isn’t the case (and the behavioral eye-tracking data didn’t have an attend and unattended condition). Thus, is possible this is just a result of more foveal luminance when the participants are focused on secondary task. It is difficult to rule this out with the current data. The authors have also reported that results were no different with a brighter background, but it’s the relative luminance issues that are the cause for concern (so this doesn’t really solve the problem). 
Re: To address this concern, we now calculated time-integrated luminance change (from background) for the hit and near-miss stimuli at different eccentricities. As shown by the figure below, the near-miss stimulus produces more luminance change (decrease) at smaller eccentricities, thus collision-sensitive activation in the foveal SC cannot be explained by the difference in luminance changes. As we discussed earlier, the collision-sensitive clusters clearly locate outside the retinotopic regions of the hit stimulus, thus cannot be explained by a retinotopic bias to the hit stimulus if there is any.

Although we didn’t compare the eye movements in attended and unattended conditions, our eye movement data in the attended condition clearly demonstrate that subjects can maintain their fixations very well during the collision detection task (see Supplementary Fig. 3b as below). Thus, the lack of collision sensitivity in the foveal SC in the attended condition is unlikely due to eye movement toward the stimuli.

In addition, there was no systematic shift in V1 retinotopic responses across eccentricities between attended and unattended, or between hit and miss conditions (as shown in the figure below), suggesting that subjects maintained fixation well in the scanner.

Further the authors don’t really have a plausible explanation for the difference between the attend and unattend condition aside from “The lack of collision sensitivity in the attended condition was likely due to the saturation of SC responses”. Therefore, there isn’t a convincing argument for the result not to have been influenced by luminance changes?
Re: The luminance change at different eccentricities was now added as a subplot in supplementary Fig.2. We highlighted in the discussion that collision sensitivity in the foveal SC was unlikely a result of difference in luminance change between hit and near-miss trajectories.

Overall I don't think the results are robust enough to warrant publication in this journal.
Re: We believe there are misunderstandings about our analysis and stimuli, we hope our explanations have sufficiently addressed all of your concerns. The main findings of collision sensitivity in the tectofugal pathways have been consistently validated across three independent fMRI experiments, including the initial 3T fMRI experiment, and the 7T fMRI experiments with healthy participants and hemianopic patients. This is rare in a single fMRI study, which usually does not have within-study replications. These reproducible results from three independent experiments should be robust enough to support our conclusions.

1. To compute the optical flow between two images, you must solve this optical flow constraint equation:

$$I_{x}u+I_{y}v+I_{t}=0$$

   - $Ix$, $Iy$, and $It$ are the spatiotemporal image brightness derivatives.
   - $u$ is the horizontal optical flow.
   - $v$ is the vertical optical flow.

   By assuming that the optical flow is smooth across the entire image, the Horn-Schunck method estimates a velocity field, $[u v]T$, that minimizes this equation:

$$E=\iint\left( I_{x}u+I_{y}v+I_{t} \right)^{2}dxdy+a\iint\{\left( \frac{\partial u}{\partial x} \right)^{2}+\left( \frac{\partial u}{\partial y} \right)^{2}+\left( \frac{\partial v}{\partial x} \right)^{2}+\left( \frac{\partial v}{\partial y} \right)^{2}\}dxdy$$

   In this equation, $\frac{\partial u}{\partial x}$ and $\frac{\partial u}{\partial y}$are the spatial derivatives of the optical velocity component, $u$, and $\alpha$ scales the global smoothness term. The Horn-Schunck method minimizes the previous equation to obtain the velocity field, $[u v]$, for each pixel in the image. This method is given by the following equations:

$$u_{x,y}^{k+1}=\bar{u}_{x,y}^{k}-\frac{I_{x}[I_{x}\bar{u}_{x,y}^{k}+I_{y}\bar{v}_{x,y}^{k}+I_{t}]}{a^{2}+I_{x}^{2}+I_{y}^{2}}$$

$$v_{x,y}^{k+1}=\bar{v}_{x,y}^{k}-\frac{I_{y}[I_{x}\bar{u}_{x,y}^{k}+I_{y}\bar{v}_{x,y}^{k}+I_{t}]}{a^{2}+I_{x}^{2}+I_{y}^{2}}$$

   In these equations, $[u_{x,y}^{k} v_{x,y}^{k}]$ is the velocity estimate for the pixel at $(x,y)$, and $[\bar{u}_{x,y}^{k} \bar{v}_{x,y}^{k}]$ is the neighborhood average of $[u_{x,y}^{k} v_{x,y}^{k}]$. For $k = 0$, the initial velocity is 0.

   To solve $u$and $v$ using the Horn-Schunck method:

   1. Compute $I$*x* and $Iy$ by using the Sobel convolution kernel, [−1 −2 −1; 0 0 0; 1 2 1], and its transposed form for each pixel in the first image.
   2. Compute $It$ between images 1 and 2 using the [−1 1] kernel.
   3. Assume the previous velocity to be 0, and compute the average velocity for each pixel using [0 1 0; 1 0 1; 0 1 0] as a convolution kernel.
   4. Iteratively solve for $u$ and $v$.
